# Non-animal-based options for animal-based foods- towards a systematic terminology

**DOI:** 10.3389/fnut.2023.1208305

**Published:** 2023-07-04

**Authors:** Nazanin Abbaspour, Ruben Sanchez-Sabate, Joan Sabaté

**Affiliations:** ^1^School of Public Health, Environmental Nutrition Research Group, Loma Linda University, Loma Linda, CA, United States; ^2^Centro de Excelencia en Psicología Económica y del Consumo (CEPEC), Núcleo Científico y Tecnológico en Ciencias Sociales y Humanidades, Universidad de La Frontera, Temuco, Chile; ^3^Núcleo de Investigación en Educación, Ciencias Sociales y Patrimonio, Universidad Adventista de Chile, Chillán, Chile

**Keywords:** alternative, substitute, replacement, analog, animal-based, plant-based

## Abstract

The market has seen a rapid increase in animal-free products intended to replace animal-based foods due to concerns for human health and environmental sustainability. However, there is a lack of consistent terminology for these products, with various terms being used interchangeably, creating ambiguity. To address this issue, we propose a systematic nomenclature that defines the most commonly used terms, namely alternative, substitute, replacement, and analog, along with examples of each. In this nomenclature, a substitute primarily serves a culinary purpose, while a replacement is concerned with nutritional properties. An analog strives to satisfy both culinary and nutritional attributes to closely mimic animal-based foods in terms of sensory, nutritional, and functional characteristics. The term “alternative” serves as an umbrella term encompassing all possibilities. This work aims to promote a clearer understanding of such products and their intended use and facilitate a unified use of terminology across disciplines. This will also enable informed decision-making for consumers and greater transparency in the food industry. The health and environmental implications of these products are not discussed in this perspective.

## Introduction

The interest in animal-free products for animal-based foods has seen a remarkable surge in recent years driven by a combination of ethical, environmental, and health considerations. With the growth of plant-based meat, dairy, and protein market, small and large companies are turning to produce a new generation of animal-free choices that imitate the taste, texture, and appearance of traditional animal-based foods (1). The industry has experienced a rapid increase in demand (2), leading to technological advancements in creating non-animal-based products, particularly plant-based, imitating animal foods (1). In 2018, the market was valued at $4.6 billion, and some projections indicate that this figure is set to reach $85 billion by 2030 (3). As the popularity and production of these products increase, so does the need for understanding and education about them. New knowledge comes with new vocabulary or new usage of the existing vocabulary. However, the wide range of terms used to describe these products has used little or no consistency and systematicity, leading to ambiguity and uncertainty about them.

**Figure 1:**
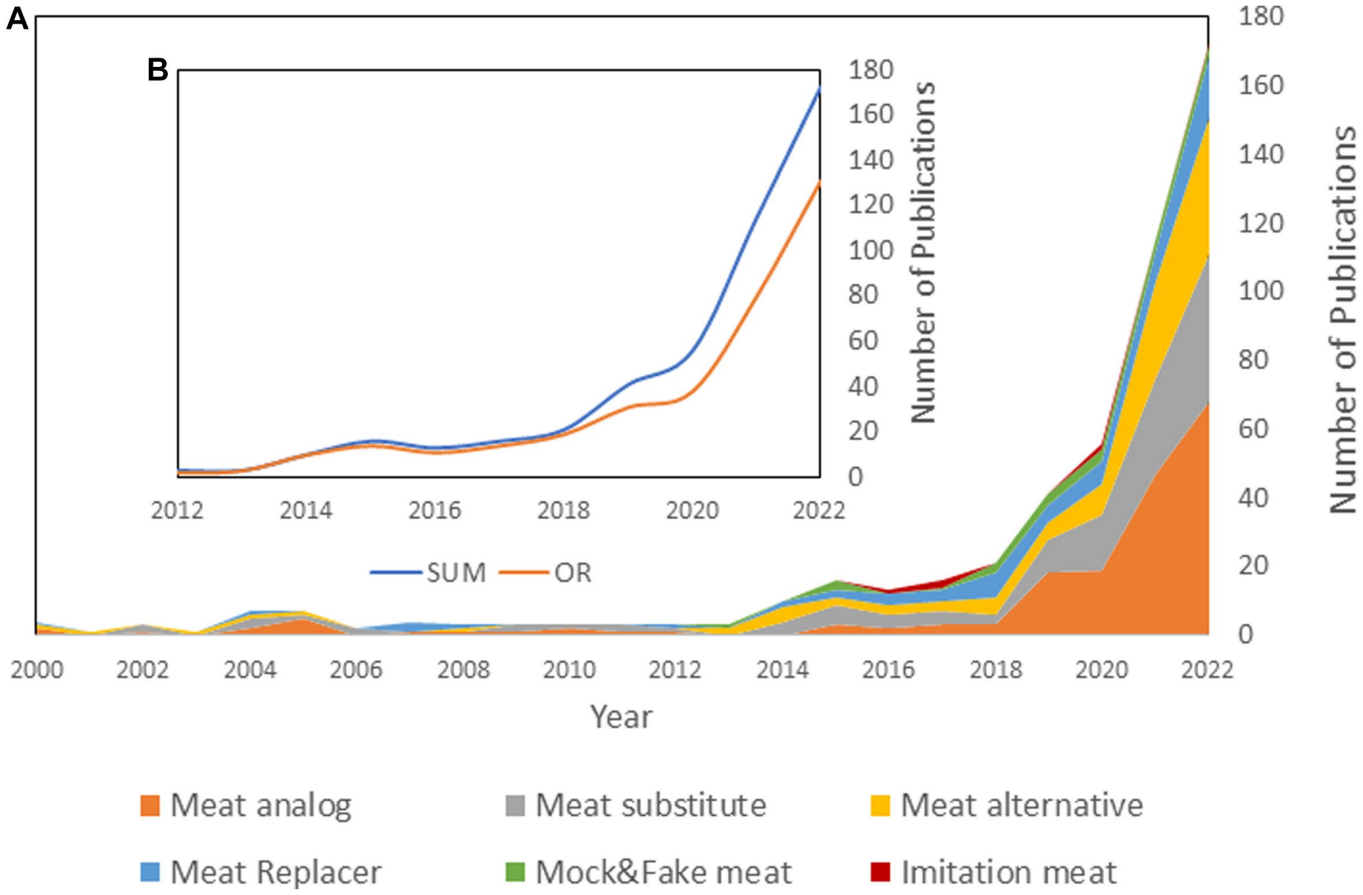
**(A)** The cumulative number of scientific publications reported by Web of Knowledge using “All Fields” containing the terms “meat alternative,” “meat analog/analogue,” “meat substitute,” “imitation meat,” “meat replacer/replacement,” and “mock/fake meat”; [**(B)**, Inset] The sum of the number of publications containing the terms “meat alternative,” “meat analog/analogue,” “meat substitute,” “imitation meat,” “meat replacer/replacement,” and “mock/fake meat” presented in **(A)** (SUM), and the number of publications that have used at least one of those terms (OR) from 2000 to 2022 reported by the Web of Knowledge using “All Fields.”

Regarding meat, our research based on the Web of Knowledge from 2000 to 2022 using “All Fields” found that the terms “meat alternative,” “meat substitute,” “meat analog or analogue,” “imitation meat,” “mock or fake meat,” and “meat replacer or replacement” have been the primary terms used. Our findings show a sharp rise in using these terms in the scientific literature over the past decade compared to their negligible use before (Figure 1A). Among different terms, “meat analog” has been increasingly used over the years, followed by “meat substitute,” “meat alternative,” and “meat replacement.” In this search formulation, some overestimation in the number of articles per term was unavoidable, as different terms may have been used within the same article. However, this does not change the remarkable increasing trend of emerging publications (4). In Figure 1B, we plotted the sum of the publication numbers for each term shown in Figure 1A and compared that with an “OR” formulation, where at least one term should exist in each publication. The difference indicates the increasing number of times several of these terms are being used within the same article. The figure shows the acceleration of this difference in the last few years. Moreover, most publications use different terms without distinction (3, 5–15). This lack of clear differentiation is also apparent in both mass and social media (15–17).

The synonymous use of words may serve some rhetorical purposes, such as enhancing readability and esthetics. However, in the realm of science, it is crucial to consistently use precise and unambiguous language to ensure clear and unified understanding. Given the abundance of non-animal-based food products available today, the lack of a distinct and purposeful differentiation represents at least a missed opportunity, calling for a well-defined language shared by different agents in the food system. In light of this, we suggest a definition for the most commonly used terms: “alternative,” “substitute,” “replacement,” and “analog,” along with examples of the products they represent. Such standardization is not only important for researchers, producers, and regulators in terms of health, food production, and labeling, but distinguishing between different options facilitates informed decision-making.

## Terminology: foundation and definitions

### Foundation

There are different dietary approaches to reducing the consumption of animal foods. The most common is choosing whole plant foods rich in protein or using other protein sources, such as plant-based meat/dairy, algae, fungi (e.g., mycoprotein), insects, or cultured meat. The plant-based products considered here entail algae and fungi as plant-like foods or ingredients. However, other options, such as insects and cultured meat, are not considered plant-based and, therefore, not discussed.

Before addressing the basis of the proposed terminology, it should be noted that the increasing demand for plant-based products as an option to animal foods is relevant in societies that primarily consume animal-based foods, such as the Western world. However, in communities where plant foods form the staple diet and animal products are consumed only occasionally, the idea of plant-based “alternatives” would be irrelevant.

The nutritional and culinary attributes are used as the basis for our definitions of the most commonly used terms, namely “substitute,” “replacement,” “analog,” and “alternative.”

The nutritional attribute considers the nutrients present in the plant-based products compared to their animal food counterpart when a nutritionally comparable plant-based option is sought for an animal-based food. Therefore, the selection is made based on the nutritional profile, which refers to the presence and quantity of key nutrients. In determining nutritional similarity, the key nutrients of a particular food, such as calcium in dairy or protein, iron, and zinc in meat, serve as the point of reference. The goal is a nutritionally comparable plant-based option to the animal-based counterpart.

The culinary attribute applies to artisanal, household, and industrial food preparation and production. The point of reference here would be the function of food, often used as an ingredient, as well as the sensory characteristics of the end product or the product itself if consumed alone. As for function in a recipe, the plant-based ingredient used in place of the animal food should have the same functional properties (e.g., thickening, foaming, emulsifying, stabilization, and gelling ability) to produce an end product with the same or very similar sensory qualities (2).

### Proposed terminology

#### Substitute

A plant-based food or ingredient that can substitute the original animal-sourced food or ingredient outside or within a recipe. When used in a recipe, a substitute should be similar, if not identical, in its culinary properties to the original ingredient. As the physiochemical and biological properties of plant-based foods notably vary from those of animal foods, it is important to find those that match the functional attributes of the animal source ingredients to produce the same or similar end product. Therefore, understanding the fundamental qualities of both the original and the substituting ingredients is necessary. However, when substituting an animal-based food with a plant-based food outside a recipe, the gastronomic and sensory aspects take precedence over functionality. In both cases, the nutritional qualities are either not considered or become secondary.

##### Examples

*- Bean patties:* vegetarian or vegan burger patties made primarily from mashed beans, such as black beans, kidney beans, or chickpeas, along with other ingredients such as vegetables, grains, and spices. Bean burgers do not try to mimic the taste or texture of meat. Moreover, although they can be a good source of protein, they have an overall different nutrient profile than meat. However, they can be prepared to have a similar texture to beef burgers with similar cooking methods, such as grilling or pan-frying. In addition, they can be served on a bun with toppings such as lettuce, tomato, onion, cheese, and condiments such as ketchup or mustard, providing a similar sensory and gastronomic experience to a traditional beef burger.

*- Plant-based milks* (e.g., nut milks, oat milk, coconut milk, hemp milk): the nutritional values of these products vary from dairy milk (18), except for soymilk; however, they typically have physicochemical and sensory properties similar to cow’s milk (1). Therefore, they can provide a culinary function and sensory experience similar to dairy milk. For example, they can substitute dairy milk in a “latte” or hot chocolate or be consumed alone. Nevertheless, some plant-based milks may not react the same way as dairy milk in certain cases, such as those that require heating and curdling of milk (1).

*- Vegetable oil:* they can be used as a substitute for butter in baking and/or cooking. Like butter, they contribute to tenderness.

*- Nuts:* although relatively high in fats (mainly unsaturated) and protein, they have a different overall nutrient composition than meat. Nevertheless, they can provide similar functionality and sensory properties as meat in various recipes. Walnuts, for example, can substitute meat to make walnut balls instead of meatballs. Similarly, nut loaves can replace meat loaves.

*- Aquafaba:* can be used as a substitute for egg white in baking (19). It is the water in which chickpeas and other legumes are cooked and has similar foaming and binding abilities to egg white (19).

*- Agar agar:* can be used as a substitute for gelatin in dishes like jellies, puddings, custard, and fruit gummies. It is extracted from red algae and has very similar gelling and stabilizing abilities as animal-sourced gelatin (20).

#### Replacement

Refers to a plant-based option with similar nutritional properties to its animal-based counterpart. The focus here is the key nutrients in the animal-based food or ingredient, and the functional and sensory attributes are secondary considerations. Therefore, the primary concern is the nutritional profile when seeking a plant-based replacement.

##### Examples

*- Tofu:* a minimally processed product made from soybeans that provides high-quality plant-based protein similar to animal protein (21). Although tofu’s protein content is lower than meat, it is often used to replace animal protein due to its high quality and digestibility. It is also considered a reasonable source of some key nutrients such as calcium and iron (22).

*- Tempeh:* another soy product made from partially cooked, fermented soybeans (21). It is dense and chewy and can be used in stir-fries, burritos, sandwiches, soups, and other dishes. Tempeh is less processed than texturized vegetable protein (TVP) and tofu. However, fermentation improves its protein digestibility and mineral bioavailability compared to tofu (23), resulting in nutritional values compatible with meat. While 100 g of beef (ground, 85% lean meat / 15% fat, patty, cooked, broiled) contains about 26 g protein, 15.4 g lipids, 18 mg calcium, 2.6 mg iron, and 6.3 mg zinc, 100 g of tempeh contains about 20 g of protein, 11 g lipids, 111 mg calcium, 2.7 mg iron, and 1.1 mg zinc (24). Although the nutrient composition of tempeh can vary depending on the brand, it provides equivalent amounts, and sometimes more, of the key nutrients such as protein, fat, iron, and calcium and, therefore, can be used as a replacement for meat.

#### Analog

Refers to a plant-based product that intends to match both the nutritional and culinary attributes of its animal food counterpart. The aim is to re-create the original animal food in terms of appearance, texture, flavor, mouthfeel, and other sensory qualities while meeting its nutritional and functional properties. Thus, their production often requires extensive processing with a careful selection of ingredients and technologies compared to “substitutes” and “replacements.”

##### Examples

*- Soymilk:* nutritionally, it is the closest to dairy milk (18, 25). It is the only plant-based milk with comparable amounts of protein, minerals, and vitamins to cow’s milk (18). Additionally, it is often fortified with vitamins and minerals, such as vitamin D and calcium, to be nutritionally compatible with dairy milk (26, 27). Despite its “beany flavor” (27), it is placed under the analog category as it can also be used as a dairy substitute, therefore meeting both the nutritional and culinary criteria.

*- First-generation meat analogs:* plant-based food products designed to mimic the texture, taste, and appearance of meat. They were developed in the 1960s and 1970s and are typically made from soy, wheat, nuts, or products such as TVP. Their production was to meet the dietary needs of vegetarians and vegans who wanted to consume a meat-like product without consuming animal-based foods. However, the initial meat analogs produced through low-level processing techniques were criticized for their lack of taste and texture compared to real meat. Some examples include veggie burgers, different forms of vegetarian chicken such as nuggets and patties, vegetarian sausages or links, hot dogs, and cold cuts. Some famous producing brands include Worthington, Yves, MorningStar Farms, Loma Linda Foods, Lightlife, Tofurky, and Gardenburger.

*- Second-generation meat analogs:* a type of non-animal-based meat that aims to replicate the texture, flavor, and appearance of real meat more closely than earlier-generation products. Unlike the first-generation meat analogs, which were simply made from soy protein, wheat gluten, or nuts, second-generation meat analogs often use a combination of plant-based or plant-like ingredients, such as mycoprotein, and food technologies to mimic meat in its entirety. They require a larger number of additives and ingredients, a higher level of processing, and extensive technological advancements (28–30). Some examples include Beyond Meat and Impossible Foods. These products utilize soy, pea, and wheat as the primary protein source (31), along with coconut oil, potato starch, and other ingredients to create products that not only taste and cook like animal meat, but also resemble its nutrient profile (1).

#### Alternative

In the context of the present perspective, an alternative refers to a food option that does not attempt to replicate its animal-based counterpart’s nutritional and culinary qualities. An alternative is simply a different choice that may have similar physical characteristics to the original animal-based food, such as texture or form (e.g., fluid or solid), and is gastronomically desirable to the consumer. However, it is not required to be equivalent in all other properties. Hence, as the definition of the word implies, it encompasses all possible options or “alternatives,” making it the broadest category.

##### Examples

- Drinking apple juice instead of milk.

- Using avocado and nuts instead of cheese and salami as part of a charcuterie board.

- An entrée of pasta and vegetables instead of beef stew.

## Few notes to consider

Although sparse literature was found using the terms fake meat and/or mock meat, we recommend refraining from using these expressions as they could be considered derogatory. As we progress from alternative to replacement or substitute to analog, there is an increase in technological requirements, inputs, and processing (e.g., edamame/beans < tofu/bean patties < Beyond Meat). Therefore, the criteria required to be met also become more stringent. While an alternative is not required to meet any specific criteria to replace the original animal food, except for being gastronomically satisfying to the consumer, the substitute must have comparable culinary properties, and the replacement must meet the nutritional qualities. The analogs, on the other hand, are expected to satisfy both nutritional and culinary characteristics and provide the same or very similar sensory experience.

Despite the industry’s efforts, some nutritional properties of analogs remain different. For example, animal foods contain mainly saturated fat, while plants primarily have unsaturated fat and no cholesterol. In the case of meat, some food companies have incorporated plant-based sources of saturated fat, such as coconut oil, into their plant-based meat products to simulate the characteristics of real meat (1). Meanwhile, a plant-based analog that is identical to its animal food counterpart in all aspects has yet to be produced. Whether these efforts are desirable from the health and environmental perspectives is beyond the scope of this perspective.

**Figure 2:**
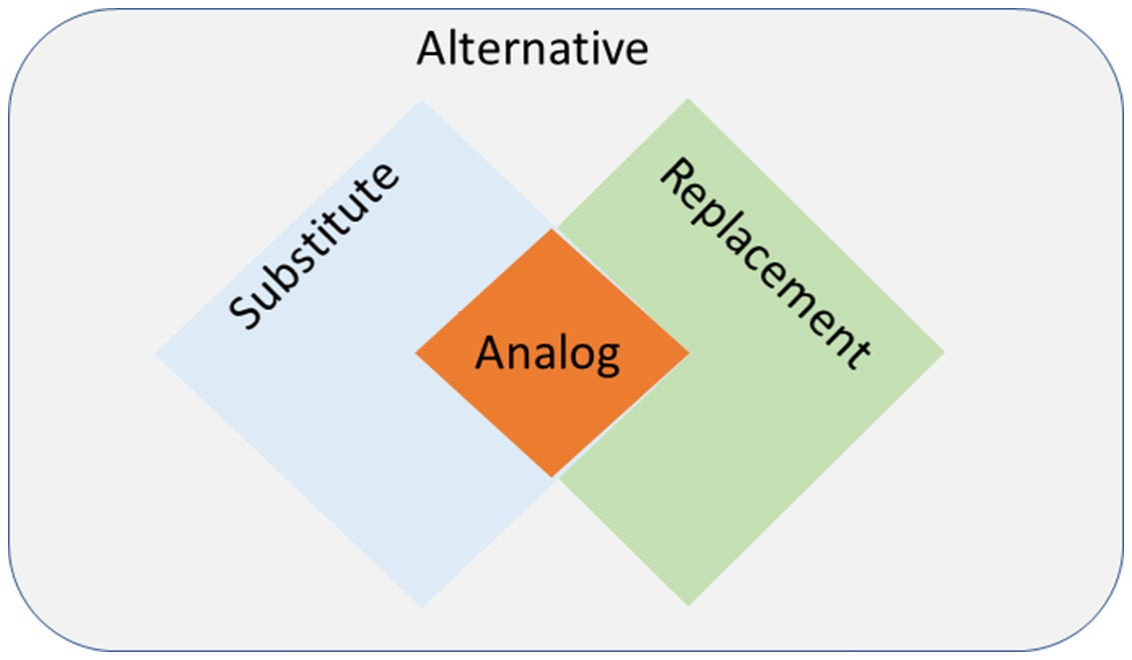
The overlapping nature of different terms describing various animal-free options for animal foods. The “alternative” is an all-inclusive term, “substitute” (culinary properties) and “replacement” (nutritional properties) overlap to give rise to “analog,” which intends to imitate animal foods in terms of sensory qualities while meeting their nutritional and functional properties.

It is important to note that the definitions presented here are not always mutually exclusive and can overlap (Figure 2). For instance, an analog can serve as a substitute, replacement, or alternative, but the reverse is not always the case. The term alternative has often been used in different platforms to refer to analogs (10); however, in precise terminology, it is simply a voluntary food choice and does not necessitate to fulfill the nutritional or culinary attributes of animal-sourced food. As the criteria for this category are not stringent, it encompasses a wide range of options, from those emulating the original food (analogs) to those that bear no resemblance. For instance, an alternative to meat can range from avocado, which only shares the physical characteristic of being solid, to tofu, which additionally meets some nutritional qualities, to second-generation meat analogs (e.g., Beyond Meat), which is the closest to meat not only in physical appearance and nutrient content, but also the sensory properties. Analogs, on the other hand, are innovative creations that require advanced technologies and are gaged by their all-aspect equivalence with the original food. It is worth re-mentioning that there is a varying degree of similarity to the original animal food regarding the nutritional and culinary characteristics among analog products, as it is a complex endeavor. However, their intention of production and use, often followed by a higher level of processing, places them in this category.

## Discussion

Systematizing the nomenclature of animal food alternatives refers to organizing and standardizing the currently used names to describe them. Our goal is to make the existing terminology consistent and clear across different disciplines, cultures, and sectors. The current ambiguity in nomenclature is best highlighted in the definition of meat alternative given by Wikipedia: “A meat alternative or meat substitute (also called plant-based meat or fake meat, sometimes pejoratively) is a food product made from vegetarian or vegan ingredients, eaten as a replacement for meat” (32). The scientific literature also abounds with such statements (5, 7, 8). To present a brief review of the recent works, Knaapila et al. (33) state that food products crafted from protein-rich, non-animal sources, designed to resemble meat and be used instead of meat are commonly known as meat analogs, meat substitutes, or meat alternatives. While these terms are often used interchangeably in the literature (34), there can be variations in their specific definitions among different authors. In recent studies focusing on the production of such products using extrusion technology, the term meat analog has frequently been employed (31, 35–40). Some define meat analogs as replacers of meat and meat products in their functionality while being similar in terms of sensory properties, particularly taste, aroma, and texture, as well as nutritional value (41–43). Kumar et al. (44) define meat analog as “a food product that approximates the esthetic qualities and/or chemical characteristics of certain types of meat.” Fiorentini et al. (8) state that “plant-based products with meat-like sensory attributes are often referred to as meat analogs, plant-based, or imitation meat.” Banerjee et al. (45) state meat analogs are also imitation meat, since they imitate the esthetic qualities of regular animal meat in terms of texture, flavor, and appearance. Meat substitutes have been defined by Elzerman et al. (46) as products specifically developed to be consumed “instead” of meat. On the other hand, they defined meat alternatives as other products that are commonly consumed as protein sources in vegetarian meals, such as pulses and nuts. However, Choudhury et al. (47) considered plant-based meat alternatives as sustainable protein sources that can replicate “the taste, texture, color, and nutritional profile of specific types of meat.”

Based on these studies, it is evident that a consensus regarding the terminology for these products has not been universally established (33). Therefore, to promote clarity and efficient communication within the plant-based food industry and among scientists, nutritionists, health professionals, consumers, and social media, we found it timely and appropriate to offer clear definitions for the commonly used terminology. This will help reduce confusion and improve understanding of the various plant-based options.

Sha et al. (4) have suggested that adhering to terms such as “meat alternative” rather than “meat analog” “would better serve the purpose of delivering sustainable protein supply,” as plant-based protein products are unlikely to replace regular meat and poultry products. The authors argue that “by doing so, the industry would avoid many of the controversies and obstacles generated from the practice of mimicking animal meat and eliminate unnecessary consumer expectations. This approach would allow scientists and food processors to focus on the development of the best possible organoleptic and nutritious qualities of food from sustainable plant proteins to feed the ever-increasing global population.” However, as “meat analog” is the most commonly used term, it would be impractical to eliminate it from the existing terminology. Moreover, despite the predicted increase in meat consumption (48), the rise in the production of these plant-based products is projected to continue as a response to their increasing demand (3). Additionally, the proposed definition of analog here considers both “organoleptic” and “nutritious” qualities, therefore, meeting the concern of the authors.

Moreover, we acknowledge the initiative by Plant Based Foods Association (PBFA) (49) to develop voluntary standards for labeling plant-based meats, milk, and yogurt in the United States. For meat, these labels include referencing the types of meat (e.g., meat, hamburger, sausage, chicken, pork) in terms of their flavor, texture, or style of preparation, the form or the type they take (e.g., nuggets, tenders, burger, patties), and qualifiers that indicate if the product is plant-based (i.e., consists mainly of ingredients derived from plants and does not contain animal ingredients of any kind), vegetarian (i.e., consists mainly of ingredients derived from plants but may contain small amounts of animal-derived ingredients, such as eggs or milk, but does not contain meat from any animal), or vegan (i.e., does not contain animal ingredients of any kind). While this information is necessary on a label, and we add that it should also contain the various additives, a systematic nomenclature is also needed at a higher level of classification that distinguishes between different terms.

In addition to our efforts, some plant-based food companies are working to standardize their product labeling to improve consumer comprehension and bolster marketing. Overall, systematizing the nomenclature for animal food alternatives is an ongoing process aimed at enhancing the precision and consistency of the terminology and facilitating greater understanding and transparency within different sectors.

Finally, the primary objective of this article is to enhance precision and consistency in the description of food components, fostering a shared understanding and transparency across various sectors. It is crucial to acknowledge that the dynamic nature of the food industry continually introduces new products to the market. As a result, our established terminology may not necessarily provide an unconditional fit for emerging and innovative food options. While this work serves as a valuable foundation, ongoing efforts are required to adapt and evolve the terminology to encompass these growing food products. By embracing the industry’s dynamic nature and promoting continuing dialog and research, we can strive to ensure accurate and effective communication in the realm of food components.

## Data availability statement

The original contributions presented in the study are included in the article/supplementary material, further inquiries can be directed to the corresponding author.

## Author contributions

NA and RS-S developed the content, drafted and edited the manuscript, and share the first authorship, JS developed the concept and content and edited the manuscript. All authors contributed to the article and approved the submitted version.

## Conflict of interest

The authors declare that this work was conducted without any commercial or financial relationships that could be construed as a potential conflict of interest.

## Publisher’s note

All claims expressed in this article are solely those of the authors and do not necessarily represent those of their affiliated organizations, or those of the publisher, the editors and the reviewers. Any product that may be evaluated in this article, or claim that may be made by its manufacturer, is not guaranteed or endorsed by the publisher.

## References

[1] McClements DJ Grossmann L. The science of plant-based foods: constructing next-generation meat, fish, milk, and egg analogs. Compr Rev Food Sci F. (2021) 20:4049–100. doi: 10.1111/1541-4337.12771, PMID: 34056859

[2] McClements DJ Grossmann L. A brief review of the science behind the design of healthy and sustainable plant-based foods. NPJ Sci Food. (2021) 5:17. doi: 10.1038/s41538-021-00099-y, PMID: 34083539 PMC8175702

[3] Santo RE Kim BF Goldman SE Dutkiewicz J Biehl EMB Bloem MW. Considering plant-based meat substitutes and cell-based meats: a public Health and food systems perspective. Front Sustain Food Sys. (2020) 4:134. doi: 10.3389/fsufs.2020.00134/full

[4] Sha L Xiong YL. Plant protein-based alternatives of reconstructed meat: science, technology, and challenges. Trends Food Sci Technol. (2020) 102:51–61. doi: 10.1016/j.tifs.2020.05.022

[5] Maningat CC Jeradechachai T Buttshaw MR. Textured wheat and pea proteins for meat alternative applications. Cereal Chem. (2021) 99:37–66. doi: 10.1002/cche.10503

[6] Alcorta A Porta A Tárrega A Alvarez MD Vaquero MP. Foods for plant-based diets: challenges and innovations. Foods. (2021) 10:293. doi: 10.3390/foods10020293, PMID: 33535684 PMC7912826

[7] Ismail BP Senaratne-Lenagala L Stube A Brackenridge A. Protein demand: review of plant and animal proteins used in alternative protein product development and production. Animal Front. (2020) 10:53–63. doi: 10.1093/af/vfaa040, PMID: 33391860 PMC7759735

[8] Fiorentini M Kinchla AJ Nolden AA. Role of sensory evaluation in consumer acceptance of plant-based meat analogs and meat extenders: a scoping review. Foods. (2020) 9:1334. doi: 10.3390/foods9091334, PMID: 32971743 PMC7555205

[9] Svarc PL Jensen MB Langwagen M Poulsen A Trolle E Jakobsen J. Nutrient content in plant-based protein products intended for food composition databases. J Food Compos Anal. (2022) 106:104332. doi: 10.1016/j.jfca.2021.104332

[10] van Vliet S Kronberg SL Provenza FD. Plant-based meats, human Health, and climate change. Front Sustain Food Syst. (2020) 4:128. doi: 10.3389/fsufs.2020.00128

[11] Priya RK Rawson A Vidhyalakshmi R Mohan RJ. Development of vegan sausage using banana floret (Musa Paradisiaca) and jackfruit (*Artocarpus heterophyllus* lam.) as a meat substitute: evaluation of textural, physico-chemical and sensory characteristics. J Food Process Pres. (2022) 46:e16118. doi: 10.1111/jfpp.16118

[12] Yuan X Jiang W Zhang D Liu H Sun B. Textural, sensory and volatile compounds analyses in formulations of sausages analogue elaborated with edible mushrooms and soy protein isolate as meat substitute. Foods. (2021) 11:52. doi: 10.3390/foods11010052 35010178 PMC8750815

[13] Duthoo E De Reu K Leroy F Weckx S Heyndrickx M Rasschaert G. To culture or not to culture: careful assessment of metabarcoding data is necessary when evaluating the microbiota of a modified-atmosphere-packaged vegetarian meat alternative throughout its shelf-life period. BMC Microbiol. (2022) 22:34. doi: 10.1186/s12866-022-02446-9, PMID: 35078415 PMC8788083

[14] Curtain F Grafenauer S. Plant-based meat substitutes in the flexitarian age: an audit of products on supermarket shelves. Nutrients. (2019) 11:2603. doi: 10.3390/nu11112603, PMID: 31671655 PMC6893642

[15] USA Today (2021). Available at: https://eu.usatoday.com/story/money/food/2022/01/20/meatless-restaurants-saved-700-k-animals-2021/6570343001/ (accessed April 9, 2023).

[16] Women’s Health (2022). Available at: https://vegworldmag.com/top-3-delicious-replacements-for-your-favorite-meat-dishes/ (accessed April 9, 2023).

[17] VegWorld, (2022). Available at: https://vegworldmag.com/top-3-delicious-replacements-for-your-favorite-meat-dishes/ (accessed April 9, 2023).

[18] Walther B Guggisberg D Badertscher R Egger L Portmann R Dubois S. Comparison of nutritional composition between plant-based drinks and cow’s milk. Front Nutr. (2022) 9:9. doi: 10.3389/fnut.2022.988707, PMID: 36386959 PMC9650290

[19] Buhl TF Christensen CH Hammershøj M. Aquafaba as an egg white substitute in food foams and emulsions: protein composition and functional behavior. Food Hydrocoll. (2019) 96:354–64. doi: 10.1016/j.foodhyd.2019.05.041

[20] Jaswir I Alotaibi A Jamal P Octavianti F Lestari W Hendri R. Optimization of extraction process of plant-based gelatin replacer. Int Food Res J. (2016) 23:2519–24.

[21] Kołodziejczak K Onopiuk A Szpicer A Poltorak A. Meat analogues in the perspective of recent scientific research: a review. Foods. (2022) 11:105. doi: 10.3390/foods11010105, PMID: 35010232 PMC8750317

[22] Eze NM Okwume UG Eseadi C Udenta EA Onyeke NG Ugwu EN. Acceptability and consumption of tofu as a meat alternative among secondary school boarders in Enugu state, Nigeria: implications for nutritional counseling and education. Medicine (Baltimore). (2018) 97:e13155. doi: 10.1097/MD.0000000000013155 30407343 PMC6250531

[23] Samtiya M Aluko RE Puniya AK Dhewa T. Enhancing micronutrients bioavailability through fermentation of plant-based foods: a concise review. Fermentation. (2021) 7:63. doi: 10.3390/fermentation7020063

[24] USDA, Food Data Central (2019). Available at: https://fdc.nal.usda.gov/ (accessed April 9, 2023).

[25] Berardy A Rubín-García M Sabaté J. A scoping review of the environmental impacts and nutrient composition of plant-based milks. Adv Nutr. (2022) 13:2559–72. doi: 10.1093/advances/nmac098, PMID: 36083996 PMC9930689

[26] Collard KM McCormick DP. A nutritional comparison of Cow’s Milk and alternative Milk products. Acad Pediatr. (2020) 21:1067–9. doi: 10.1016/j.acap.2020.12.007 33373745

[27] Vanga SK Raghavan V. How well do plant-based alternatives fare nutritionally compared to cow’s milk? J Food Sci Technol. (2018) 55:10–20. doi: 10.1007/s13197-017-2915-y, PMID: 29358791 PMC5756203

[28] Schmid E-V Farahnaky A Adhikari B Torley PJ. High moisture extrusion cooking of meat analogs: a review of mechanisms of protein texturization. Comp Rev. (2020) 21:4573–609. doi: 10.1111/1541-4337.13030, PMID: 36120912

[29] Zahari I Östbring K Purhagen JK Rayner M. Plant-based meat analogues from alternative protein: a systematic literature review. Foods. (2022) 11:2870. doi: 10.3390/foods11182870, PMID: 36140998 PMC9498552

[30] Wittek P Karbstein HP Emin MA. Blending proteins in high moisture extrusion to design meat analogues: rheological properties, morphology development and product properties. Foods. (2021) 10:1509. doi: 10.3390/foods10071509, PMID: 34209076 PMC8307526

[31] Kyriakopoulou K Keppler JK van der Goot AJ. Functionality of ingredients and additives in plant-based meat analogues. Foods. (2021) 10:600. doi: 10.3390/foods10030600, PMID: 33809143 PMC7999387

[32] Wikipedia. Available at: https://en.wikipedia.org/wiki/Meat_alternative (accessed April 9, 2023).

[33] Knaapila A Michel F Jouppila K Sontag-Strohm T Piironen V. Millennials’ consumption of and attitudes toward meat and plant-based meat alternatives by consumer segment in Finland. Foods. (2022) 11:456. doi: 10.3390/foods11030456, PMID: 35159606 PMC8834568

[34] Thavamani A Sferra TJ Sankararaman S. Meet the meat alternatives: the value of alternative protein sources. Curr Nutr Rep. (2020) 9:346–55. doi: 10.1007/s13668-020-00341-1, PMID: 33151486

[35] Guo Z Teng F Huang Z Lv B Lv X Babich O. Effects of material characteristics on the structural characteristics and flavor substances retention of meat analogs. Food Hydrocoll. (2020) 105:105752. doi: 10.1016/j.foodhyd.2020.105752

[36] Kendler C Duchardt A Karbstein HP Emin MA. Effect of oil content and oil addition point on the extrusion processing of wheat gluten-based meat analogues. Foods. (2021) 10:697. doi: 10.3390/foods10040697, PMID: 33805896 PMC8064384

[37] Saldanha do Carmo C Knutsen SH Malizia G Dessev T Geny A Zobel H. Meat analogues from a faba bean concentrate can be generated by high moisture extrusion. Future Foods. (2021) 3:100014. doi: 10.1016/j.fufo.2021.100014

[38] Schreuders FKG Sagis LMC Erni P BI Boom RM van der Goot AJ. Mapping the texture of plant protein blends for meat analogues. Food Hydrocoll. (2021) 118:106753. doi: 10.1016/j.foodhyd.2021.106753

[39] Sun C Ge J He J Gan R Fang Y. Processing, quality, safety, and acceptance of meat analogue products. Engineering. (2021) 7:674–8. doi: 10.1016/j.eng.2020.10.011

[40] Ferawati F Zahari I Barman M Hefni M Ahlström C Witthöft C. High-moisture meat analogues produced from yellow pea and faba bean protein isolates/concentrate: effect of raw material composition and extrusion parameters on texture properties. Foods. (2021) 10:843. doi: 10.3390/foods10040843, PMID: 33924424 PMC8070665

[41] Dekkers BL Boom RM van der Goot AJ. Structuring processes for meat analogues. Trends Food Sci Technol. (2018) 81:25–36. doi: 10.1016/j.tifs.2018.08.011

[42] Collier ES Oberrauter LM Normann A Norman C Svensson M Niimi J. Identifying barriers to decreasing meat consumption and increasing acceptance of meat substitutes among Swedish consumers. Appetite. (2021) 167:105643. doi: 10.1016/j.appet.2021.105643, PMID: 34389377

[43] Prabha K Ghosh P S A Joseph RM Krishnan R Rana SS. Recent development, challenges, and prospects of extrusion technology. Future Foods. (2021) 3:100019. doi: 10.1016/j.fufo.2021.100019

[44] Kumar P Chatli MK Mehta N Singh P Malav OP Verma AK. Meat analogues: health promising sustainable meat substitutes. Crit Rev Food Sci Nutr. (2017) 57:923–32. doi: 10.1080/10408398.2014.939739, PMID: 25898027

[45] Banerjee S Rao A Zahid SA. Meat analogue: a short review on processing aspects. Act Sci Nutr Health. (2021) 5:77–81. Available at: https://www.actascientific.com/ASNH/pdf/ASNH-05-0835.pdf

[46] Elzerman JE Keulemans L Sap R Luning PA. Situational appropriateness of meat products, meat substitutes and meat alternatives as perceived by dutch consumers. Food Qual Prefer. (2021) 88:104108. doi: 10.1060/j.foodqual.2020.104108

[47] Choudhury D Singh S Seah JSH Yeo DCL Tan LP. Commercialization of plant-based meat alternatives. Trends Plant Sci. (2020) 25:1055–8. doi: 10.1016/j.tplants.2020.08.006 32896491

[48] OECD/FAO. OECD-FAO Agricultural Outlook 2019-2028. Paris: OECD Publishing (2019).

[49] Plant Based Foods Association (PBFA) (2021). Available at: (https://www.plantbasedfoods.org/wp-content/uploads/PBFA-Labeling-Standards-for-Meat-Alternatives.pdf).

